# A case-control study on the risk factors associated with the occurrence of non-tuberculous mycobacteria pulmonary disease in bronchiectasis patients

**DOI:** 10.1186/s12890-023-02768-y

**Published:** 2023-11-20

**Authors:** Yinping Feng, Jing Guo, Shuirong Luo, Zunjing Zhang

**Affiliations:** Department of infectious diseases, Lishui Hospital of traditional Chinese Medicine Affiliated to Zhejiang University of traditional Chinese Medicine, Lishui, 323000 China

**Keywords:** Bronchiectasis, Non-tuberculous mycobacteria, Risk factors

## Abstract

**Objective:**

The objective of this study is to analyze the risk factors associated with bronchiectasis combined with non-tuberculous mycobacteria pulmonary disease(NTM-PD) and provide a basis for more effective prevention and treatment strategies.

**Methods:**

The study subjects for this manuscript were patients with bronchiectasis who were admitted to the infection department between January 2021 and June 2023.There were 34 patients with NTM-PD in the observation group, and 52 patients with simple bronchiectasis in the control group. Basic information, imaging features, serum albumin levels, and infection indicators were collected from both groups of patients.Univariate and multivariate logistic regression analysis were performed to analyze the risk factors for NTM-PD in patients with bronchiectasis.

**Results:**

Multivariate logistic regression analysis revealed that bronchiectasis exacerbation occurring at least twice a year(OR = 3.884, 95% CI: 1.200-12.568), involvement of three or more lung lobes with bronchiectasis (OR = 3.932, 95% CI: 1.208–12.800), hypoalbuminemia (OR = 3.221, 95% CI: 1.015–10.219), and the NLR index (OR = 1.595, 95% CI: 1.200-2.119) were significant risk factors for non-tuberculous mycobacteria pulmonary disease in individuals with bronchiectasis (P < 0.05).

**Conclusion:**

Patients with bronchiectasis accompanied by NTM-PD present specific risk factors that should be promptly addressed through prevention and treatment.

## Introduction

Bronchiectasis(BCS) is a chronic respiratory disease characterised by a clinical syndrome of cough, sputum production, and bronchial infection, Radiologically,it is identified by abnormal and permanent dilatation of the bronchi [1]. The prevalence in Europe and North America ranges from 67 to 556 per100,000, and can be as high as 1200 per 100,000 in China [2].Infection is the most significant factor among the various causes of bronchiectasis. Notably, there has been an increasing number of non-tuberculous mycobacteria (NTM) isolated from the sputum or alveolar lavage fluid of bronchiectasis patients. Most studies (81%) shown declining incidence rates of tuberculosis. However, the proportion of mycobacterial disease caused by NTM has been rising in almost every geographic area (94%) [3]. Furthermore, bronchiectasis and non-tuberculous mycobacterium pulmonary disease (NTM-PD) interact to exacerbate the disease. This study aims to explore the risk factors associated with NTM-PD in patients with bronchiectasis, providing a theoretical basis for early prevention and treatment.

## Object and methods

### Research object selection

Patients with bronchiectasis who were hospitalized in our department from January 2021 to June 2023 were selected as the study population. Out of these, 52 patients had simple bronchiectasis and 34 patients had NTM-PD.This study was approved by the Lishui Traditional Chinese Medicine Hospital (Approval number: 2023LW-041).

## Methods

### Patients’ inclusion and exclusion criteria

Inclusion criteria: (1) Patients who meet the diagnostic criteria for bronchiectasis, as outlined in the “European Respiratory Society guidelines for the management of adult bronchiectasis” [1]; (2) Patients who meet the diagnostic criteria for NTM-PD, as described in the “British Thoracic Society guidelines for the management of non-tuberculous mycobacterial pulmonary disease (NTM-PD)” [4]. (3) Patients with complete clinical data.

Exclusion criteria: (1) Patients with incomplete clinical data; (2) Patients with other severe organ dysfunctions and chronic wasting diseases, such as tumors.

### Patients data collection

Demographic characteristics such as age, gender, and ethnicity, as well as body mass index(BMI), were collected. The annual number of acute exacerbations, the number of lung lobes involved, imaging data, blood albumin levels, and the NLR index(= Neutrophils (*10^9/L)/Lymphocytes (*10^9/L))were also included in the data collection. Comorbidities were also documented.

### Case grouping

Patients were divided into two groups:the bronchiectasis group and the bronchiectasis group with NTM-PD.

### Identification techniques for Non-tuberculosis Mycobacterium

Non-tuberculous Mycobacteria strains were identified using colloidal gold method for MGIT960 liquid culture-positive strains.The NTM strains were further characterized using 16 S rRNA and hsp65 gene sequencing,as well as gene chip analysis.

#### Hypoalbuminemia

It refers to plasma albumin level of < 35 g/L.

#### Imaging diagnostic criteria

The use of unified 64 slice spiral CT (GE, LightSpeed, VCT) for scanning is recommended. The parameters used include a layer thickness of 0.625 mm, an interval of 10 mm, a voltage of 120 kV, bone algorithm reconstruction, and HRCT for image acquisition. The diagnosis of bronchiectasis can be made if the diameter of the bronchus/accompanying pulmonary artery is greater than 1.

Diagnostic criteria for acute exacerbation of bronchiectasis are based on the British Thoracic Society Guideline for bronchiectasis in adults [5]:cough, changes in sputum volume, purulent sputum, difficulty breathing or exercise tolerance, fatigue or discomfort, and hemoptysis. The presence of three or more of these six symptoms worsening for more than 48 h is considered necessary for clinical intervention by healthcare professionals.

### Statistical analysis

All data were statistically analyzed using SPSS 25.0 software. Metric data that followed a normal distribution were represented by mean ± standard deviation ($\left( {\bar \chi \pm s} \right)$), and intergroup comparisons were performed using independent sample t-tests. Metric data that did not follow a normal distribution were represented by median (M) and interquartile range (IQR,P25, P75), and intergroup comparisons were performed using Mann-Whitney U-tests. Counting data are presented as (n,%) and were used for intergroup comparison inspection. Multivariate logistic regression analysis was used to identify the risk factors for bronchiectasis accompanied by NTM-PD, and statistical significance was determined at P < 0.05.

## Results

### Case screening

**Fig. 1:**
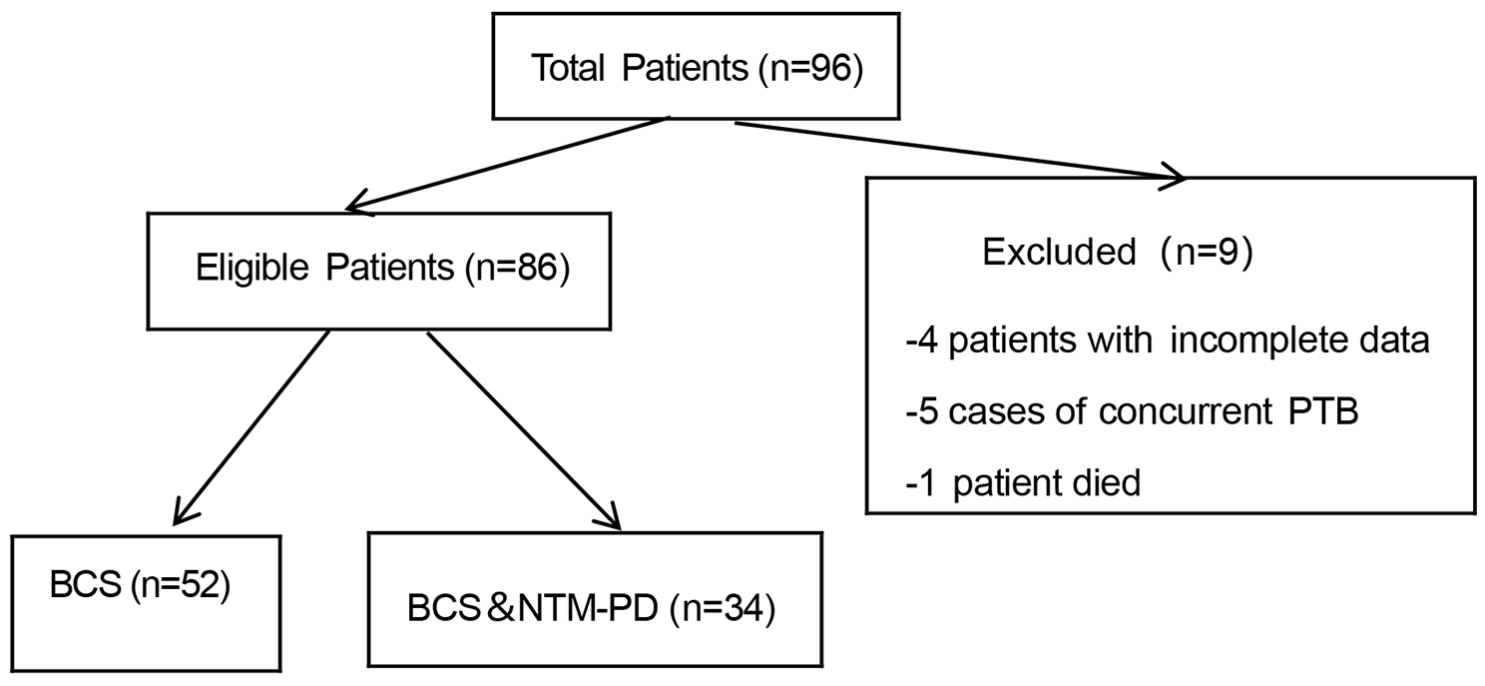
A flow chart for screening eligible studies

A total of 96 hospitalized patients diagnosed with bronchiectasis were included in this study. Four of these patients had incomplete clinical data, five were also diagnosed with tuberculosis, and unfortunately, one patient passed away during the course of the study. After excluding these cases, a total of 86 patients were ultimately included in the analysis. Among them, 34 patients had concurrent NTM-PD, while 52 patients had simple bronchiectasis (Fig. 1).

### Single factor analysis of NTM-PD in patients with bronchiectasis

There were no statistically significant differences in age and gender distribution between the two groups (P > 0.05). Univariate analysis showed no statistically significant differences in body mass index, smoking, cavities, and diabetes between patients with bronchiectasis accompanied by NTM-PD and patients with simple bronchiectasis (P > 0.05). Patients with bronchiectasis accompanied by NTM-PD had acute exacerbations occurring twice or more per year and three or more bronchiectasis lobes, as well as a statistically significant difference in hypoalbuminemia compared to patients with simple bronchiectasis (P < 0.05). Furthermore, the inflammation index NLR was also significantly different between the two groups(Table 1).

**Table 1:** Single factor analysis of bronchiectasis accompanied by NTM-PD

Characteristic	BCS(n = 52)	BCS&NTM-PD (n = 34)	Statistical value	*P*
Age[$\left( {\bar \chi \pm s} \right)$]	64.6 ± 14.5	66.4 ± 10.8	0.630	0.530
Gender[n(%)]				
Female	25(48.1)	16(47.1)		
Male	27(51.9)	18(52.9)	0.009	0.926
BMI [M(P25, P75)]	19.4(17.6,21.0)	18.5(15.6,21.1)	1.462	0.144
Smoking [n(%)]				
No	37(71.2)	27(79.4)		
Yes	15(28.8)	7(49.1)	0.736	0.391
Acute exacerbation[n(%)]				
<2	40(76.9)	14(41.2)		
≥ 2	12(23.1)	20(58.8)	11.244	0.001
Number of lobes[n(%)]				
<3	37(71.2)	11(32.4)		
≥ 3	15(28.8)	23(67.6)	12.550	<0.001
Pulmonary cavity				
No	32(61.5)	15(44.1)		
Yes	20(44.1)	19(55.9)	2.517	0.113
Hypoalbuminemia[n(%)]				
No	39(75.0)	12(35.3)		
Yes	13(25.0)	22(64.7)	13.429	<0.001
Diabetes[n(%)]				
No	32(61.5)	19(55.9)		
Yes	20(38.5)	15(44.1)	0.273	0.602
NLR [M(P25, P75)]	2.6(1.8,3.5)	4.4(2.9,7.0)	3.984	<0.001

### Multivariate Logistics regression analysis of bronchiectasis with NTM-PD

The statistically significant factors identified in the single factor analysis were included as independent variables in constructing the multi-factor logistics regression equation (Table 2 ). The results indicated that patients with bronchiectasis who experienced acute exacerbations ≥ 2 times per year (OR = 3.884, 95%CI:1.200-12.568),had ≥ 3 bronchiectasis lung lobes (OR = 3.932, 95%CI:1.208–12.800), hypoalbuminemia (OR = 3.221, 95%CI:1.015–10.219) and a high NLR index (OR = 1.595, 95%CI:1.200-2.119) were at increased risk for NTM-PD(Table 3). The significance level for all associations was set at P < 0.05.

**Table 2:** Assignment of categorical variables in logistic regression analysis

Serial number	Independent variable	Assign
X1	acute exacerbation ≥ 2 twice a year	0 = No, 1 = Yes
X2	Branched lung lobes ≥ 3	0 = No, 1 = Yes
X3	Hypoalbuminemia	0 = No, 1 = Yes
X4	NLR index	Continuous variable

**Table 3:** Multivariate Logistic Regression Analysis of Bronchiectasis Accompanied by NTM-PD

Variable	*β*	*SE*	Wald *χ²*	*P*	*OR*	95%*CI*
acute exacerbation ≥ 2 twice a year	1.357	0.599	5.128	0.024	3.884	1.200-12.568
Branched lung lobes ≥ 3	1.369	0.602	5.169	0.023	3.932	1.208–12.800
Hypoalbuminemia	1.170	0.589	3.942	0.047	3.221	1.015–10.219
NLR index	0.467	0.145	10.350	0.001	1.595	1.200-2.119
Constant	4.023	0.856	22.070	< 0.001	0.052	

## Discussion

In recent years, both the incidence and prevalence of bronchiectasis have increased. In the United States, the prevalence of adult bronchiectasis is approximately 139 per 100,000 individuals [6]. In the United Kingdom, the prevalence of adult bronchiectasis was 525.8 per 100,000 in 2013 [7]. The pathogenesis of bronchiectasis is mainly characterized by airway remodeling and dilation caused by chronic inflammation, with infection being the primary cause. Among these infections, the presence or colonization of *Pseudomonas aeruginosa* is closely associated with the development and progression of bronchiectasis [8]. With the advancement of molecular biological detection technology, the number of bronchiectasis patients with NTM infection has also significantly increased. The prevalence of NTM isolation in patients with bronchiectasis ranges from 2–63% [9, 10]. While clinical studies on NTM infection in patients with bronchiectasis mainly focus on imaging findings, clinical symptoms, and strain identification, there is limited research on the risk factors of NTM-PD in these patients. Because NTM are primarily found in patients with chronic respiratory diseases such as bronchiectasis or silicosis because they are environmental pathogens and facultative intracellular parasites [11]. One study suggested that in patients with bronchiectasis, the impairment physical and immune barriers of the airway wall lead to a decrease in the function of phagocytic cells in airway secretions, resulting in opportunistic NTM infections due to insufficient secretion of lysozyme [12]. Additionally, there is evidence indicating that the occurrence of NTM disease in patients with bronchiectasis may be related to α1-antitrypsin deficiency [13]. In conclusion, structural abnormalities can impair mucociliary clearance, leading to chronic infection, and perpetuating a vicious cycle [14]. Immune-mediated inflammatory response has been confirmed as the main mechanism underlying this cycle [15]. Bronchiectasis and NTM-PD mutually influence each other, although the specific cause and effect relationship has yet to be clearly determined.

The objective of this study was to investigate the risk factors of NTM-PD in patients with bronchiectasis. Clinical data and imaging data from the study participants were collected for univariate statistical analysis. The factors that showed statistical significance were then included in a multivariate logistic regression analysis to construct equations that identified the risk factors contributing to the development of NTM-PD in patients with bronchiectasis. The results showed that patients with bronchiectasis who experienced two or more acute exacerbations per year were more likely to develop NTM-PD (OR = 3.884, 95%CI:1.200-12.568). The number of acute exacerbations in the previous year is included in the Bronchiectasis Severity Index (BSI) and is one of the indicators in the Exacerbations-Frequency, Age, Colonization, Extension, and Disease Extent (E-FACED) score scale [16]. This suggests that a higher number of acute exacerbations is associated with increased damage to the airway wall and mucosal barrier, thus increasing the likelihood of opportunistic NTM infection. Chest imaging also revealed that patients with bronchiectasis affecting more than three lobes were more likely to have NTM infection (OR = 3.932, 95%CI:1.208–12.800). Additionally, chest imaging of patients with NTM-PD typically showed manifestation such as bronchiectasis, tree in bud signs, cavities, and nodules [17]. The distribution of CT lesions in NTM-PD patients primarily involved both lungs, with bronchiectasis being most commonly observed in the middle lobe of right lung and the lingula of the left lung [18]. Mycobacterium avium complex (MAC) was the most frequently identified NTM species, followed by M. abscessus [19]. As the number of lung lobes affected by bronchiectasis lesions increases, airway obstruction and reduced ventilation may result,leading to more frequent acute exacerbations, more severe clinical manifestations, worsening structural lung disease,and increased likelihood of NTM infection. Patients with bronchiectasis combined with hypoalbuminemia were also more likely to develop NTM-PD (OR = 3.221, 95%CI:1.015–10.219). Hypoalbuminemia is closely related to malnutrition, and studies have shown that it is an independent risk factor for bronchiectasis combined with NTM infection. This is because NTM is a conditional pathogen with low virulence, and hypoalbuminemia increases the risk of NTM infection Schweitzer et al. [20] have also demonstrated that nutritional status plays an important role in the development of NTM-PD. In recent years, the NLR has been widely used as an indicator of systemic inflammatory response and has shown predictive value in evaluating respiratory diseases, including bronchiectasis associated with infection [21]. Neutrophils participate in the inflammatory response, while lymphocytes protect endothelial cells and help control inflammation. Therefore, the balance between inflammation activation and control can be reflected in the NLR,with a higher ratio indicating a more severe inflammatory response [22]. Prolonged elevation of NLR level suggests a long-term, severe, and uncontrolled immune response and progressive systemic inflammatory response, which may contribute to the worsening of patient comorbidities and adverse outcomes [23]. In patients with bronchiectasis, NLR can also be used to assess the severity of acute exacerbation [24]. This study also found that NLR levels were significantly increased in patients with bronchiectasis combined with NTM-PD (OR = 1.595, 95%CI:1.200-2.119),, indicating that NLR is one of the risk factors associated with NTM-PD in these patients.

## Conclusion

In conclusion,acute exacerbations of bronchiectasis occurring at least twice per year, the presence of three or more bronchiectasis lung lobes, hypoalbuminemia, and an elevated inflammatory index known as the NLR, are more likely to be accompanied by NTM-PD.,Early intervention is necessary to reduce the prevalence rate and improve the cure rate. However, it is important to note that this study is retrospective in nature and has a limited sample size, which introduces certain limitations. It is hoped that future prospective studies with larger sample sizes can be designed to further explore the risk factors associated with NTM-PD in patients with bronchiectasis. Such studies will provide a more solid foundation for effective prevention and treatment strategies.

## Data Availability

All data generated or analysed during this study are included in this article. Further enquiries can be directed to the corresponding author.
